# Knowledge, attitude, and practice of orthopedic surgery patients regarding the prevention and treatment of venous thromboembolism

**DOI:** 10.3389/fpubh.2026.1676207

**Published:** 2026-02-09

**Authors:** Jie Wu, Xuefen Li, Ying Hao

**Affiliations:** 1 Department of Pulmonary and Critical Care Medicine, Affiliated Hospital of Inner Mongolia Medical University, Hohhot, Inner Mongolia, China; 2 Department of Pharmacy, Inner Mongolia Autonomous Region Hospital of Traditional Chinese Medicine, Hohhot, Inner Mongolia, China; 3 Health Management Center, Affiliated Hospital of Inner Mongolia Medical University, Hohhot, Inner Mongolia, China

**Keywords:** knowledge, attitude, practice, orthopedic surgery, patient education, structural equation modeling, venous thromboembolism

## Abstract

**Objective:**

To assess the current status of knowledge, attitude, and practice (KAP) regarding the prevention and treatment of venous thromboembolism (VTE) among orthopedic surgery patients.

**Methods:**

A cross-sectional study was conducted between January and October, 2024, in the Inner Mongolia region at a single center. A self-developed structured questionnaire (informed by relevant VTE guidelines and prior KAP studies) was used to collect demographic information and KAP scores related to VTE prevention and treatment. Descriptive statistics, group comparisons, correlation analysis, and structural equation modeling were conducted.

**Results:**

A total of 545 orthopedic surgery patients participated in the study. Among the respondents, 279 (51.19%) were male, and 252 (46.24%) reported a prior history of VTE. Additionally, 326 patients (59.82%) reported using anticoagulant medications for thrombosis prevention. The knowledge, attitude, and practice scores were 4.48 ± 2.37 (possible range: 0–12), 30.28 ± 5.64 (possible range: 8–40), and 23.65 ± 5.54 (possible range: 6–30), respectively. In the correlation analysis, significant positive correlations were found between knowledge and attitude (r = 0.128, *p* = 0.003), as well as attitude and practice (r = 0.448, *p* < 0.001), respectively. Structural equation modeling showed that attitude was positively associated with practice (standardized *β* = 0.907, *p* = 0.002).

**Conclusion:**

Orthopedic surgery patients demonstrated insufficient knowledge but generally positive attitude and proactive practice concerning the prevention and treatment of VTE. Enhancing patient education on VTE prevention and management should be prioritized to bridge the knowledge gap, leveraging their positive attitude to improve compliance with evidence-based practice.

## Introduction

Venous thromboembolism (VTE), which includes deep vein thrombosis (DVT) and pulmonary embolism (PE), is a severe and potentially life-threatening condition (1). DVT is characterized by abnormal blood coagulation within deep veins, leading to venous reflux disorders and chronic venous insufficiency, with symptoms including lower limb pain, swelling, and impaired mobility (2). In severe cases, thrombus detachment can result in PE, a critical complication with potentially fatal consequences (3, 4).

The incidence of VTE is alarmingly high among long-term bedridden patients, reaching up to 31%, a rate significantly exceeding that observed in non-bedridden individuals (5). This research is particularly crucial for clinicians and patients in China, where orthopedic surgery volumes are rapidly increasing, yet standardized VTE prevention awareness remains limited. Orthopedic surgery patients are at heightened risk for VTE due to prolonged immobility, surgical trauma, and systemic inflammatory responses, making it among the most devastating complications in this field (6–8). Because orthopedic procedures frequently involve the lower limbs and prolonged postoperative immobility, patients’ knowledge, attitudes, and practices regarding VTE prevention are especially critical compared with general surgical populations (9).

Understanding patients’ knowledge, attitude, and practice (KAP) regarding VTE prevention and management is critical for mitigating these risks. The KAP survey, a widely used diagnostic tool in health literacy research, operates on the principle that increased knowledge fosters positive attitude, which subsequently shape behaviors. However, effective prevention and treatment of VTE require not only clinical interventions but also patient education and adherence (10, 11). Identifying gaps in knowledge, addressing misconceptions, and overcoming barriers to adherence can help improve clinical outcomes, enhance patient-centered care, and reduce VTE-related morbidity and mortality (12). This study applies a KAP framework to identify gaps in patients’ understanding and behaviors that may influence VTE prevention efforts.

Despite extensive research focusing on healthcare professionals’ awareness and management strategies for VTE (13–16), studies specifically targeting the KAP of orthopedic surgery patients remain scarce, particularly in the context of China. This gap in the literature underscores the need for this study, which aims to assess the current status of KAP among Chinese orthopedic surgery patients regarding VTE prevention and treatment.

## Materials and methods

### Study design and participants

A single-center cross-sectional study was conducted between January and October 2024 at the Affiliated Hospital of Inner Mongolia Medical University, a tertiary hospital in Hohhot, Inner Mongolia, China, among patients who had undergone orthopedic surgery. The study was approved by the Ethics Committee of the Affiliated Hospital of Inner Mongolia Medical University (Approval No. KY2023098), and informed consent was obtained from all participants prior to their inclusion in the research.

Participants were enrolled based on the following inclusion criteria: they must have undergone orthopedic surgery, be at least 18 years old, possess the cognitive ability to understand the study’s objectives and voluntarily provide informed consent, and be conscious and able to complete the questionnaire independently. Cognitive ability was assessed through a brief screening process where participants were asked to demonstrate understanding of the study’s purpose by explaining it back to the research team in their own words.

### Questionnaire introduction

The questionnaire used in this study was developed based on a comprehensive review of prior literature and adherence to relevant domestic and international guidelines (17–19). This was a self-developed questionnaire rather than a direct adaptation of an existing scale, and its item pool was generated with reference to relevant VTE guidelines and previously published KAP questionnaires in public health research. To ensure the content validity of the instrument, the initial draft underwent a rigorous evaluation by three experts in respiratory and critical care medicine, whose feedback was incorporated to enhance the instrument’s rigor. Subsequently, a pilot survey involving 26 participants was conducted to assess the questionnaire’s reliability and validity. Pilot participants’ baseline characteristics, including age distribution, gender, education level, residence, comorbidities, and surgical types, have now been provided in the Supplementary Table 1. The overall Cronbach’s *α* was 0.827, indicating high internal consistency. Confirmatory factor analysis (CFA) was performed to further evaluate construct validity, and the corresponding model fit indices (CFI = 0.968, TLI = 0.965, IFI = 0.968, RMSEA = 0.035) are reported in Supplementary Table 2. In this study, CFA was used to examine whether the retained items fit the intended three-domain KAP structure (knowledge, attitude, and practice), and the results supported this structure. The finalized questionnaire, administered in Chinese, comprises four distinct sections: demographic information, knowledge, attitude, and practice (Supplementary material). The knowledge dimension consists of 12 items, each scored as 1 point for a correct response and 0 points for an incorrect or unclear response, yielding a total score range of 0 to 12. The attitude dimension includes 8 items assessed using a 5-point Likert scale ranging from “Strongly Agree” (5 points) to “Strongly Disagree” (1 point), resulting in a total score range of 8–40. Finally, the practice dimension encompasses 6 items, with responses ranging from “Never” (1 point) to “Always” (5 points), producing a total score range of 6–30. To facilitate the interpretation of results, thresholds were established for each dimension: scores equal to or exceeding 70.0% of the total score were defined as indicative of adequate knowledge, positive attitude, and proactive practice (20). This threshold was adopted based on previous KAP studies that similarly defined adequate knowledge, positive attitude, and proactive practice using a ≥70% cut-off (20–22).

### Sample size calculation

To determine the required sample size for a cross-sectional study, the following formula is used: *n* = (Z^2^ × P × (1 - P))/E^2^.

n is the required sample size; Z is the *z*-value corresponding to the desired confidence level (for a 95% confidence level, *Z* = 1.96); P is the estimated proportion of the population (commonly assumed to be 0.5 when unknown); E is the margin of error (typically set at 0.05). For a 95% confidence level, with *p* = 0.5 and E = 0.05, the formula calculates as: *n* = (1.96^2^ × 0.5 × (1–0.5)) / 0.05^2^ ≈ 385. Considering a potential 20% non-response rate, the target sample size was increased to 482 participants.

### Questionnaire distribution and quality control

This study employed a digital approach to questionnaire distribution by utilizing QR codes, which facilitated online completion. The distribution was conducted across orthopedic departments within the hospital. During the study period, eligible orthopedic surgery patients in the participating departments were invited to complete the QR-code–based questionnaire, and 545 patients ultimately participated in the survey. To ensure data accuracy and prevent non-eligible participants from completing the survey, each QR code was linked to a unique access code that was only provided to patients who met the inclusion criteria after verification of their surgical records. Participants were given the option to complete the questionnaire by scanning the QR code directly or through their involvement in a patient education group, which served as an additional channel for engagement. However, the use of a digital QR-code survey may have introduced selection bias, as patients with limited digital literacy or without access to smartphones may have been underrepresented. For patients with lower literacy levels, trained research assistants provided one-on-one explanations using simplified language and visual aids to ensure comprehension. To enhance clarity and address any potential misunderstandings, a designated individual was available to provide explanations and guidance regarding the questionnaire, thereby ensuring the accuracy and reliability of the responses.

### Statistical methods

Statistical analyses were conducted using SPSS 27.0 and AMOS 26.0 (both from IBM, Armonk, NY, USA). The normality of score distributions for each dimension was assessed using the Shapiro–Wilk test. Continuous variables with a normal distribution were reported as means ± standard deviation (SD). For comparisons between two groups, the independent samples t-test was used for normally distributed data, while the Wilcoxon-Mann–Whitney test was applied for non-normally distributed data. When comparing three or more groups, analysis of variance (ANOVA) was conducted for normally distributed data with homogeneity of variance; the Kruskal-Wallis test was used for non-normally distributed data or when variance was not homogeneous. The Spearman correlation coefficient assessed the relationships among the KAP dimensions. To examine the interactions between knowledge, attitude, and practice within the theoretical framework, structural equation modeling (SEM) was utilized, estimating both direct and indirect effects to evaluate mediation pathways. Model fit was evaluated using multiple indices, including the root mean square error of approximation (RMSEA < 0.08), standardized root mean square residual (SRMR < 0.08), Tucker-Lewis index (TLI > 0.8), and comparative fit index (CFI > 0.8) in line with commonly used SEM guidelines (23, 24). For the purpose of logistic regression analysis, the practice score was dichotomized into ‘proactive practice’ (score ≥ 21, i.e., 70% of the maximum score) and ‘less proactive practice’ (score < 21). Furthermore, multivariable logistic regression models were conducted to identify independent factors associated with proactive practice. Variables with a *p*-value < 0.05 in the univariate analysis were considered for inclusion in the multivariable model. For the SEM, standardized path coefficients were estimated along with their 95% confidence intervals using bootstrap resampling procedures (5,000 iterations). A two-sided *p*-value of less than 0.05 was considered statistically significant.

## Results

### Basic information on the population

In total, 570 questionnaires were initially collected. We excluded 13 questionnaires due to abnormal BMI values and 12 questionnaires from participants younger than 18 years old, resulting in 545 valid questionnaires included in the final analysis. Out of 545 Orthopedic surgery patients who participated in this study, 279 (51.19%) were male, with mean age of 55.10 ± 13.48 years, 286 (52.48%) were residing in rural areas, 222 (40.73%) had education of high school/secondary school, 284 (52.11%) had an average monthly income per capita of less than 2,000 Yuan, and 252 (46.24%) had a history of venous thromboembolism. 326 (59.82%) had medication to prevent thrombosis. The surgical categories were distributed as follows: 163 (29.91%) underwent arthroscopic surgery, 108 (19.82%) had joint replacement, 91 (16.70%) received fracture fixation, and 183 (33.58%) underwent other procedures including soft tissue repairs, ligament reconstructions, and hardware removals. The mean knowledge, attitude, and practice scores were 4.48 ± 2.37 (possible range: 0–12), 30.28 ± 5.64 (possible range: 8–40), and 23.65 ± 5.54 (possible range: 6–30) (Table 1).

**Table 1:** Baseline characteristics.

Variables	*N* (%)	Knowledge		Attitude		Practice	
		**Mean ± SD**	**Statistics/P**	**Mean ± SD**	**Statistics/P**	**Mean ± SD**	**Statistics/P**
Total	545	4.48 ± 2.37		30.28 ± 5.64		23.65 ± 5.54	
Gender			−0.810/0.418		−0.693/0.488		1.220/0.223
Male	279 (51.19)	4.89 ± 2.47		30.43 ± 5.55		23.62 ± 5.39	
Female	266 (48.81)	4.79 ± 2.27		30.12 ± 5.74		23.67 ± 5.70	
Age (years old)	55.10 ± 13.48						
Residence			0.120/0.942		21.474/<0.001		0.859/0.651
Rural	286 (52.48)	4.80 ± 1.85		29.35 ± 5.57		23.46 ± 5.66	
Urban	189 (34.68)	4.88 ± 2.97		31.25 ± 5.92		23.47 ± 5.85	
Suburban	70 (12.84)	4.89 ± 2.51		31.44 ± 4.46		24.87 ± 3.86	
Education			2.065/0.559		45.376/<0.001		7.079/0.069
Middle school or below	161 (29.54)	4.75 ± 2.28		28.88 ± 6.50		22.63 ± 6.20	
High school/vocational school	222 (40.73)	4.98 ± 1.73		30.03 ± 4.94		23.94 ± 4.90	
Associate degree	92 (16.88)	4.84 ± 2.01		30.72 ± 4.83		24.34 ± 5.18	
Bachelor’s degree or above	70 (12.85)	4.61 ± 4.16		33.74 ± 5.20		24.14 ± 6.11	
Average monthly income per capita			0.164/0.921		38.561/<0.001		1.518/0.468
<2,000	284 (52.11)	4.85 ± 1.81		29.57 ± 5.45		23.56 ± 5.52	
2,000–5,000	194 (35.60)	4.87 ± 2.28		30.18 ± 5.69		23.61 ± 5.50	
>5,000–10,000	67 (12.29)	4.70 ± 4.15		33.58 ± 5.20		24.10 ± 5.83	
Type of medical insurance			2.859/0.414		7.105/0.069		3.443/0.328
Only social medical insurance	350 (64.22)	4.77 ± 2.45		30.30 ± 5.80		23.65 ± 5.57	
Only commercial medical insurance	112 (20.55)	5.07 ± 1.75		29.21 ± 5.71		22.98 ± 6.04	
Both social and commercial medical insurance	64 (11.74)	4.92 ± 2.71		31.42 ± 4.97		24.56 ± 4.94	
No insurance	19 (3.49)	4.53 ± 2.89		32.32 ± 2.58		24.47 ± 3.20	
BMI			3.590/0.166		1.547/0.461		8.505/0.014
<18.5	42 (7.71)	4.43 ± 2.38		31.45 ± 4.53		25.31 ± 4.42	
18.5–23.9	284 (52.11)	4.80 ± 2.36		30.05 ± 5.68		23.54 ± 5.76	
≥24.0	219 (40.18)	4.97 ± 2.40		30.36 ± 5.77		23.47 ± 5.42	
Underlying disease			8.209/0.315		42.251/<0.001		9.572/0.214
Diabetes	63 (11.56)	4.95 ± 1.92		29.71 ± 5.52		23.60 ± 5.02	
Hypertension	93 (17.06)	4.38 ± 2.01		30.37 ± 5.16		24.02 ± 4.86	
Varicose veins	75 (13.76)	5.21 ± 1.68		30.11 ± 4.76		24.20 ± 4.67	
Overweight/obesity	59 (10.83)	5.24 ± 1.87		29.85 ± 5.41		23.78 ± 5.53	
Myocardial infarction	43 (7.89)	4.93 ± 1.64		28.93 ± 6.05		22.63 ± 5.94	
Malignant tumors	41 (7.52)	4.95 ± 1.67		29.34 ± 5.73		23.37 ± 5.99	
Rheumatic and autoimmune diseases	68 (12.48)	4.78 ± 1.64		28.57 ± 6.18		22.65 ± 6.26	
None	103 (18.90)	4.64 ± 3.97		32.99 ± 5.50		24.05 ± 6.17	
Part with orthopedic surgery			8.340/0.214		9.415/0.152		11.635/0.071
Knee joint	80 (14.68)	5.09 ± 2.99		31.38 ± 6.02		23.93 ± 5.67	
Ankle joint	149 (27.34)	4.99 ± 1.94		29.64 ± 5.92		23.15 ± 5.81	
Hip joint	123 (22.57)	4.37 ± 1.92		30.02 ± 4.90		24.01 ± 5.00	
Shoulder	16 (2.94)	5.25 ± 3.17		29.63 ± 6.81		20.50 ± 6.54	
Wrist	42 (7.71)	4.57 ± 2.87		30.83 ± 5.77		23.14 ± 6.38	
Spine	56 (10.28)	5.07 ± 2.72		30.73 ± 5.49		24.66 ± 5.00	
Elbow joint	79 (14.50)	4.91 ± 2.30		30.30 ± 5.56		23.92 ± 5.27	
Type of orthopedic surgery			4.814/0.186		4.927/0.177		0.765/0.858
Arthroscopic surgery	163 (29.91)	5.07 ± 2.04		30.10 ± 5.12		23.97 ± 5.05	
Joint replacement	108 (19.82)	4.94 ± 2.02		29.60 ± 5.81		23.44 ± 5.65	
Fracture fixation	91 (16.70)	4.44 ± 2.71		30.90 ± 5.97		23.11 ± 6.12	
Others	183 (33.58)	4.77 ± 2.64		30.53 ± 5.81		23.74 ± 5.62	
Smoking habits			−0.243/0.808		3.613/<0.001		2.740/0.006
Yes	322 (59.08)	4.86 ± 1.88		29.63 ± 5.61		23.30 ± 5.48	
No	223 (40.92)	4.81 ± 2.95		31.22 ± 5.57		24.15 ± 5.60	
Drinking habits			−0.680/0.496		2.992/0.003		1.483/0.138
Yes	305 (55.96)	4.90 ± 1.91		29.63 ± 5.60		23.41 ± 5.54	
No	240 (44.04)	4.76 ± 2.86		31.11 ± 5.60		23.95 ± 5.55	
History of venous thromboembolism			−0.414/0.679		3.700/<0.001		0.951/0.342
Yes	252 (46.24)	4.93 ± 1.70		29.29 ± 5.68		23.33 ± 5.77	
No	293 (53.76)	4.76 ± 2.83		31.13 ± 5.48		23.91 ± 5.33	
Medication prevent thrombosis			−3.362/<0.001		2.043/0.041		0.311/0.756
Yes	326 (59.82)	5.14 ± 2.09		30.03 ± 5.48		23.75 ± 5.28	
No	219 (40.18)	4.39 ± 2.68		30.66 ± 5.86		23.48 ± 5.92	
Family history of thrombosis			0.948/0.343		0.340/0.733		−0.531/0.595
Yes	40 (7.34)	4.58 ± 1.96		30.05 ± 5.67		24.10 ± 5.37	
No	505 (92.66)	4.86 ± 2.40		30.30 ± 5.64		23.61 ± 5.56	
Knowledge about venous thromboembolism			45.712/<0.001		9.469/0.050		1.878/0.758
From a doctor’s explanation	165 (30.28)	5.29 ± 2.41		30.42 ± 6.12		23.57 ± 5.89	
Lectures, online or offline courses	116 (21.28)	4.79 ± 1.55		29.73 ± 5.12		23.73 ± 5.23	
Internet, public accounts, short videos	134 (24.59)	5.07 ± 2.07		29.62 ± 5.94		23.22 ± 5.73	
Books, literature	64 (11.74)	5.42 ± 1.61		30.41 ± 4.68		24.45 ± 4.51	
No knowledge	66 (12.11)	2.74 ± 3.40		32.11 ± 5.24		23.76 ± 5.78	

### KAP scores across participant characteristics

Knowledge scores differed significantly by thromboprophylactic medication use (*p* < 0.001) and sources of venous thromboembolism–related information (*p* < 0.001). Attitude scores differed significantly by education level (*p* < 0.001), place of residence (urban/suburban: 31.25–31.44 vs. rural: 29.35 ± 5.57, *p* < 0.001), average monthly income (*p* < 0.001), underlying disease status (*p* < 0.001), smoking habits (non-smokers: 31.22 ± 5.57 vs. smokers: 29.63 ± 5.61, *p* < 0.001), drinking habits (non-drinkers: 31.11 ± 5.60 vs. drinkers: 29.63 ± 5.60, *p* = 0.003), history of venous thromboembolism (no: 31.13 ± 5.48 vs. yes: 29.29 ± 5.68, p < 0.001), and thromboprophylactic medication use (no: 30.66 ± 5.86 vs. yes: 30.03 ± 5.48, *p* = 0.041). Practice scores differed significantly by body mass index (*p* = 0.014) and smoking habits (non-smokers: 24.15 ± 5.60 vs. smokers: 23.30 ± 5.48, *p* = 0.006) (Table 1). Post-hoc multiple comparison results are in Supplementary Table 3.

### Response distribution of KAP

The distribution of knowledge dimension revealed that the three knowledge items with the lowest correctness rates were as follows: “The most common site for deep vein thrombosis is the upper limb.” (K6) with 21.65%, “Basic preventive measures and physical preventive measures alone are sufficient to prevent venous thromboembolism after orthopedic surgery.” (K9) with 22.57%, and “Anticoagulant medications (e.g., heparin) administered before and after major surgeries such as joint replacement can prevent blood clots and reduce the occurrence of pulmonary embolism.” (K12) with 28.07% (Table 2).

**Table 2:** Distribution of knowledge dimension responses.

Items	**Correct *n* (%)**
1. Venous thromboembolism refers to the abnormal clotting of blood in veins, leading to complete or partial blockage of the blood vessel.	264 (48.44)
2. Patients undergoing major orthopedic surgeries (e.g., total hip or total knee replacement) are at increased risk of venous thrombosis due to factors like vascular injury and reduced activity.	390 (71.56)
3. Deep vein thrombosis and pulmonary embolism are two clinical manifestations of venous thromboembolism occurring in different locations and at different stages.	299 (54.86)
4. Pulmonary embolism is a major cause of death during the perioperative period in orthopedic surgery, and 90% of emboli originate from deep vein thrombosis.	189 (34.68)
5. Among patients with proximal deep vein thrombosis, 50% typically present with symptomatic or asymptomatic pulmonary embolism.	229 (42.02)
6. The most common site for deep vein thrombosis is the upper limb.	118 (21.65)
7. Major trauma and orthopedic surgery are risk factors for venous thromboembolism.	190 (34.86)
8. If pulmonary embolism is untreated or treatment is delayed, the mortality rate can be as high as 85%, but with active and proper treatment, the mortality rate can drop to 10%.	257 (47.16)
9. Basic preventive measures and physical preventive measures alone are sufficient to prevent venous thromboembolism after orthopedic surgery.	123 (22.57)
10. For patients with traumatic orthopedic injuries, the risk period for venous thrombosis starts immediately after injury. Thrombosis tendencies may appear within 24 h of injury, and the high-risk period for thrombosis extends to 35 days post-surgery.	271 (49.72)
11. During the acute phase of lower limb deep vein thrombosis, the affected limb should be elevated to relieve swelling, and hot compresses and massage should be applied.	154 (28.26)
12. Anticoagulant medications (e.g., heparin) administered before and after major surgeries such as joint replacement can prevent blood clots and reduce the occurrence of pulmonary embolism.	153 (28.07)

**Figure 1:**
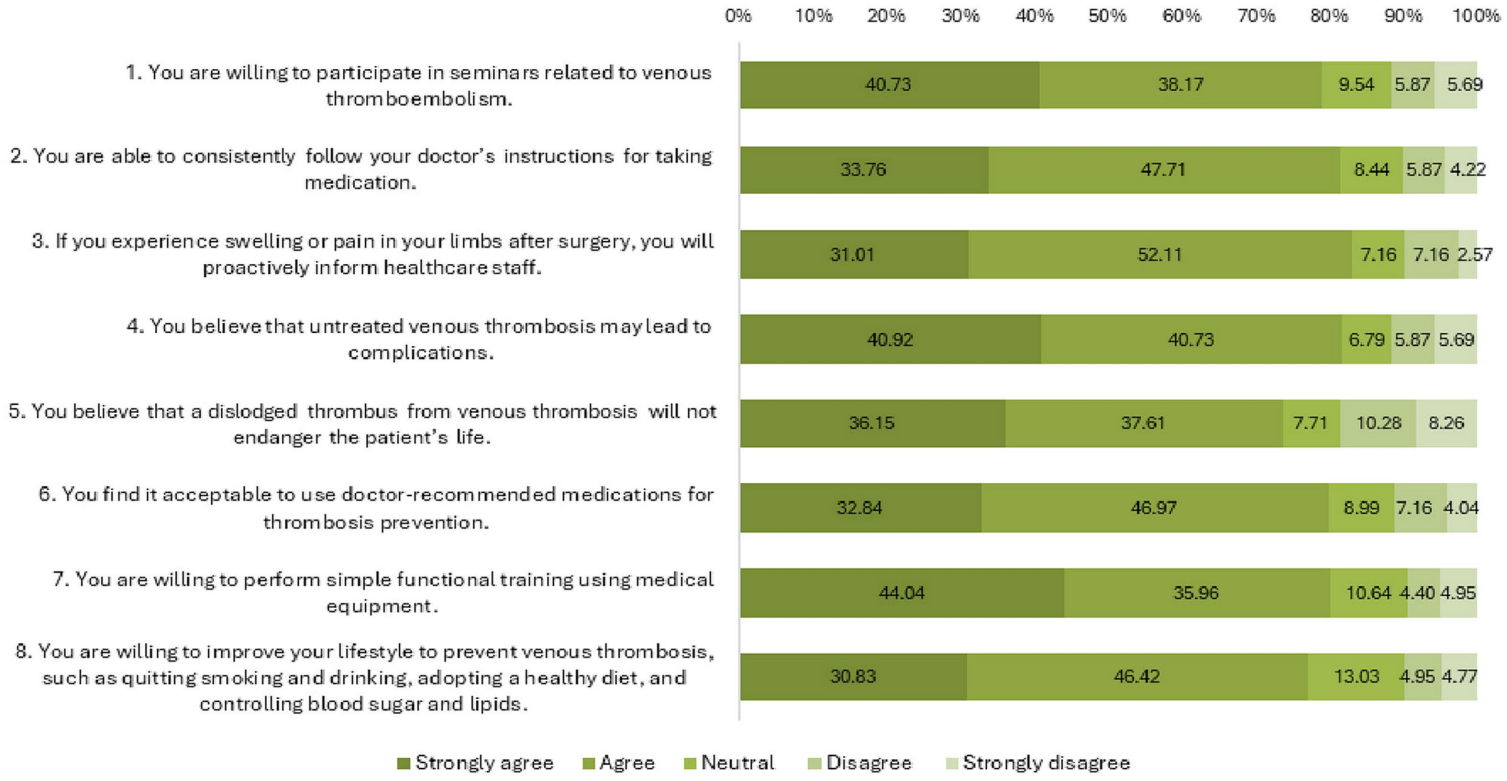
Distribution of attitude dimension responses.

**Figure 2:**
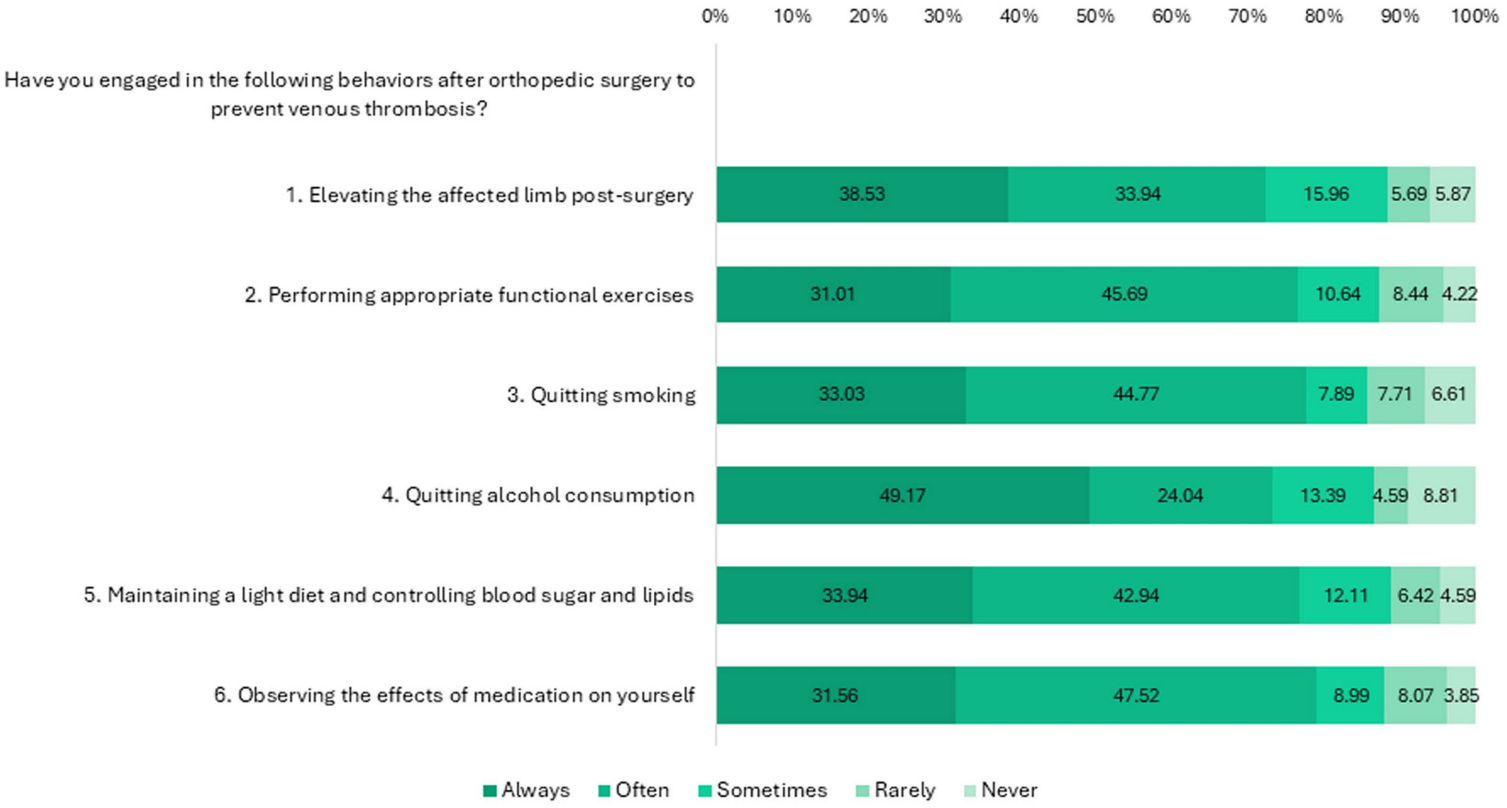
Distribution of practice dimension responses.

Responses to the attitude and practice dimensions are summarized in Figure 1 and Figure 2, respectively. Notably, a non-trivial portion of patients expressed attitudes inconsistent with clinical facts (e.g., underestimating the life-threatening nature of a dislodged thrombus) and reported suboptimal adherence to lifestyle practices like smoking and alcohol cessation.

### Correlation analysis and SEM

The correlation analysis revealed significant positive correlations: between knowledge and attitude (r = 0.128, *p* = 0.003) and between attitude and practice (*r* = 0.448, *p* < 0.001) (Table 3).

**Table 3:** Correlation analysis.

	Knowledge	Attitude	Practice
Knowledge	1		
Attitude	0.128 (P = 0.003)	1	
Practice	0.050 (P = 0.242)	0.448 (P < 0.001)	1

The fitting indices of the structural equation model (CMIN/DF = 1.659, RMSEA = 0.035, IFI = 0.968, TLI = 0.965, CFI = 0.968) all met the recommended thresholds, indicating satisfactory model fit (Supplementary Table 4). The SEM estimates showed that attitude had a significant positive direct effect on practice (standardized *β* = 0.907, 95% CI: 0.839–0.958, *p* = 0.002). Knowledge had a significant direct effect on attitude (standardized *β* = 0.110, 95% CI: 0.006–0.226, *p* = 0.034), no significant direct effect on practice (standardized *β* = −0.031, 95% CI: −0.123–0.069, *p* = 0.510), and a significant indirect effect on practice via attitude (standardized indirect *β* = 0.100, 95% CI: 0.006–0.215, *p* = 0.033) (Table 4 and Supplementary Figure 1).

**Table 4:** SEM effect estimates.

Model paths	Standardized total effects		Standardized direct effects		Standardized indirect effects	
	*β* (95%CI)	*P*	*β* (95%CI)	*P*	*β* (95%CI)	*P*
Knowledge → Attitude	0.110 (0.006,0.226)	0.034	0.110 (0.006,0.226)	0.034		
Knowledge → Practice	0.069 (−0.048,0.198)	0.216	−0.031 (−0.123,0.069)	0.510		
Attitude → Practice	0.907 (0.839,0.958)	0.002	0.907 (0.839,0.958)	0.002		
Knowledge → Practice					0.100 (0.006,0.215)	0.033

### Multivariable logistic regression analysis

The results showed that attitude score was independently associated with better preventive practice (OR = 1.282, 95% CI: 1.210–1.358, *p* < 0.001). In contrast, knowledge score was not independently associated with practice after adjustment (OR = 0.992, 95% CI: 0.906–1.086, *p* = 0.864). With regard to educational level, compared with patients with middle school education or below, those with a bachelor’s degree or above showed lower odds of engaging in proactive practice (OR = 0.253, 95% CI: 0.120–0.530, p < 0.001), whereas no significant associations were observed for high school/vocational education or associate degree education. Regarding surgical site, patients undergoing shoulder surgery were less likely to demonstrate proactive preventive practice than those undergoing knee joint surgery (OR = 0.190, 95% CI: 0.048–0.758, *p* = 0.019) (Table 5).

**Table 5:** Univariate and multivariate logistic regression analyses of factors associated with practice.

Variables	Univariate logistic regression		Multivariate logistic regression	
	OR (95%CI)	*P*	OR (95%CI)	*P*
Knowledge score	1.040 (0.966, 1.120)	0.300	0.992 (0.906–1.086)	0.864
Attitude score	1.242 (1.181, 1.306)	<0.001	1.282 (1.210–1.358)	<0.001
Gender				
Male	Ref.			
Female				
Age (years old)	1.001 (0.988, 1.014)	0.928		
Residence				
Rural	Ref.			
Urban	0.704 (0.482, 1.028)	0.069		
Suburban	1.263 (0.712, 2.242)	0.425		
Education				
Middle school or below	Ref.		Ref.	
High school/vocational school	1.156 (0.759, 1.760)	0.499	0.876 (0.529–1.451)	0.607
Associate degree	1.774 (1.010, 3.117)	0.046	1.255 (0.646–2.438)	0.502
Bachelor’s degree or above	0.788 (0.446, 1.391)	0.411	0.253 (0.120–0.530)	<0.001
Average monthly income per capita				
<2,000	Ref.			
2,000–5,000	0.857 (0.585, 1.257)	0.430		
>5,000-10,000	0.610 (0.355, 1.048)	0.074		
Type of medical insurance				
Only social medical insurance	1.661 (0.657, 4.197)	0.283		
Only commercial medical insurance	1.444 (0.543, 3.839)	0.461		
Both social and commercial medical insurance	1.980 (0.697, 5.625)	0.200		
No insurance	Ref.			
BMI				
<18.5	Ref.			
18.5–23.9	0.653 (0.315, 1.354)	0.252		
≥24.0	0.570 (0.272, 1.195)	0.137		
Underlying disease				
Diabetes	1.183 (0.616, 2.271)	0.613		
Hypertension	1.210 (0.676, 2.168)	0.521		
Varicose veins	1.197 (0.644, 2.222)	0.570		
Overweight/obesity	1.149 (0.591, 2.232)	0.682		
Myocardial infarction	0.665 (0.325, 1.363)	0.265		
Malignant tumors	1.732 (0.781, 3.840)	0.177		
Rheumatic and autoimmune diseases	1.328 (0.697, 2.529)	0.389		
None	Ref.			
Part with orthopedic surgery				
Knee joint	Ref.		Ref.	
Ankle joint	0.845 (0.480, 1.487)	0.559	0.927 (0.474–1.813)	0.824
Hip joint	1.077 (0.595, 1.949)	0.806	1.090 (0.536–2.218)	0.811
Shoulder	0.179 (0.053, 0.609)	0.006	0.190 (0.048–0.758)	0.019
Wrist	1.077 (0.489, 2.371)	0.854	1.289 (0.511–3.251)	0.591
Spine	1.346 (0.642, 2.821)	0.431	1.469 (0.612–3.528)	0.389
Elbow joint	1.098 (0.569, 2.117)	0.781	1.156 (0.534–2.503)	0.712
Type of orthopedic surgery				
Arthroscopic surgery	Ref.			
Joint replacement	1.047 (0.626, 1.751)	0.862		
Fracture fixation	0.667 (0.395, 1.128)	0.131		
Others	0.997 (0.639, 1.555)	0.989		
Smoking habits				
Yes	0.853 (0.596, 1.220)	0.384		
No	Ref.			
Drinking habits				
Yes	1.071 (0.753, 1.524)	0.702		
No	Ref.			
History of venous thromboembolism				
Yes	0.943 (0.664, 1.339)	0.742		
No	Ref.			
Medication prevent thrombosis				
Yes	1.128 (0.790, 1.611)	0.507		
No	Ref.			
Family history of thrombosis				
Yes	1.511 (0.738, 3.096)	0.259		
No	Ref.			
Knowledge about venous thromboembolism				
From a doctor’s explanation	1.163 (0.651, 2.077)	0.610		
Lectures, online or offline courses	1.400 (0.753, 2.603)	0.288		
Internet, public accounts, short videos	1.410 (0.770, 2.580)	0.266		
Books, literature	2.037 (0.973, 4.265)	0.059		
No knowledge	Ref.			

## Discussion

These findings suggest that targeted educational interventions to enhance patients’ knowledge may be linked to more favorable attitudes and practices; however, causal relationships cannot be inferred from this cross-sectional study. The findings may also inform future research and clinical practice by supporting the integration of routine KAP assessments and VTE-focused preoperative counseling into orthopedic care pathways.

The observed knowledge deficiencies, particularly concerning specific risk factors and the role of pharmacologic prophylaxis, are consistent with findings in other surgical populations (25, 26). In this study, while patients showed awareness of some general risk factors, such as the impact of orthopedic surgery on thrombosis, they lacked detailed comprehension of the pathology, common symptoms, and preventive strategies. The low knowledge scores among patients already taking anticoagulant medications (60% of participants) and those with prior VTE history (40% of participants) suggest that current educational approaches may be insufficient or poorly targeted. Similar patterns have been observed in other healthcare contexts, where knowledge gaps are prevalent among populations with lower health literacy or reduced access to educational resources (27, 28). These findings suggest a broader trend of insufficient patient education in perioperative care, particularly in areas with socio-economic disparities.

Patients’ attitudes were generally positive, consistent with studies showing that patients are often willing to follow medical advice but may lack the foundational knowledge to do so effectively (29, 30). Specifically, the most prominent knowledge gaps were reflected by the lowest-correctness items: “The most common site for deep vein thrombosis is the upper limb.” (K6), “Basic preventive measures and physical preventive measures alone are sufficient to prevent venous thromboembolism after orthopedic surgery.” (K9), and “Anticoagulant medications (e.g., heparin) administered before and after major surgeries such as joint replacement can prevent blood clots and reduce the occurrence of pulmonary embolism.” (K12).

These misconceptions may reduce vigilance for lower-limb thrombosis symptoms and may undermine adherence to evidence-based prophylaxis (particularly combined prophylaxis and anticoagulant use), with potential implications for patient safety. The observed variations in attitude between urban and rural patients, as well as between those with different education levels, align with previous findings that socio-economic status and educational attainment significantly influence health perceptions (31, 32). Patients with higher education levels or urban residency demonstrated more informed attitude, likely due to better access to health information and healthcare services.

Patients demonstrated moderate adherence to recommended behaviors for VTE prevention. While many consistently elevated affected limbs or performed functional exercises, adherence to lifestyle changes like smoking cessation and reduced alcohol consumption was less consistent. These results reflect broader trends in similar populations, where preventive practices requiring significant lifestyle adjustments are often less successfully adopted (33, 34). Moreover, healthcare system limitations, including a lack of structured follow-up programs and patient-centered counseling, may exacerbate the issue. The broader literature indicates that sustained behavioral changes require not only patient education but also continuous reinforcement through supportive healthcare frameworks (35, 36).

Correlation analyses indicated that knowledge significantly influenced attitudes, but had a limited direct impact on practice. Attitudes served as a mediator, strongly predicting patients’ engagement in preventive behaviors. Structural equation modeling confirmed this mediating effect, underscoring the importance of enhancing both knowledge and attitudes to optimize practice. In this study, the standardized path from attitude to practice was relatively strong. This may partly reflect the conceptual proximity between attitudes toward VTE prevention and self-reported preventive behaviors in a KAP framework, especially when both constructs are assessed using Likert-type items within the same questionnaire. Although attitude (8 items) and practice (6 items) were measured as distinct domains, some overlap in content emphasis (e.g., endorsement of preventive importance and reported adherence) cannot be fully excluded, which may inflate the association. In addition, SEM estimates in cross-sectional self-report data can be sensitive to shared method variance. Therefore, this pathway should be interpreted as a strong statistical association rather than a causal effect, and future studies using longitudinal designs or objective behavioral indicators are warranted to further validate the magnitude of the relationship. This pattern aligns with behavioral frameworks such as the Health Belief Model and the Theory of Planned Behavior, which emphasize that attitudes shape health-related intentions and behaviors. According to the Health Belief Model, perceived severity and perceived benefits directly affect the willingness to engage in preventive actions, suggesting that patients with positive attitudes toward VTE prevention are more likely to adopt recommended practices (37, 38). Similarly, the TPB highlights attitude as a key determinant of behavioral intention, mediated through personal evaluation of the outcomes of a health behavior. In the context of VTE prevention, patients who believe that preventive actions are beneficial and clinically meaningful are more likely to translate such attitudes into consistent practice. These theoretical perspectives reinforce our findings that attitude serves as a meaningful mediator linking knowledge acquisition to behavioral engagement in orthopedic surgery patients. Aligning with broader evidence suggesting that interventions combining educational and motivational components are most effective in enhancing patient behaviors (39, 40).

Patients with higher education levels, despite having more positive attitudes, were less likely to engage in proactive practice. Possible explanations include: (1) an unmeasured confounder (e.g., type of employment, social support) correlated with both education and practice; (2) highly educated patients may interpret “proactive practice” differently or feel capable of self-managing their recovery with less adherence to prescribed routines; or (3) healthcare providers may communicate differently, assuming a higher baseline understanding and providing less direct instruction. This finding highlights the complexity of health behaviors and suggests that education level alone is not a simple predictor of adherence.

To address these gaps, standardized VTE education should be integrated into perioperative care. At the unit level, practical strategies could include nurse-led education sessions, standardized counseling at admission and discharge, and accessible visual aids. Embedding KAP-informed education into routine workflows may improve patient engagement and adherence. Studies in similar healthcare contexts have shown that such programs significantly improve patient understanding and adherence to preventive measures (41, 42). Community-based outreach initiatives, particularly in rural areas, could further bridge knowledge gaps by making educational resources more accessible (43). Additionally, digital platforms, such as mobile health apps and online learning modules, could provide scalable solutions for delivering tailored health education (44, 45).

This study has several limitations that should be acknowledged. First, as a cross-sectional study, it only provides a snapshot of patients’ knowledge, attitude, and practice at a single point in time, limiting the ability to establish causality between variables. Second, the reliance on self-reported data introduces the potential for response bias, including possible social desirability bias, as participants may have overestimated or underestimated their actual knowledge, attitude, or practice. Third, the study was conducted in a single region (Inner Mongolia), which may restrict the generalizability of the findings to other populations with different demographic or cultural characteristics, while, as this was a cross-sectional study, the SEM findings reflect associations rather than causal relationships, and causal inferences cannot be drawn. Additionally, the use of non-probability sampling may limit the generalizability of the findings to broader orthopedic patient populations. Moreover, because data were collected using a QR-code–based online questionnaire, older, less tech-savvy, rural, or less educated patients may have been underrepresented, potentially biasing the sample toward younger, urban, and better educated individuals. This potential selection bias may limit the generalizability of the KAP estimates to the broader orthopedic surgery population, particularly in settings with a higher proportion of older or rural patients.

In addition, the relatively high proportion of participants reporting prior VTE (46.24%) suggests that the sample may include more patients with VTE-related experience than would be expected in some orthopedic settings, and the findings should therefore be extrapolated with caution.

## Conclusion

In conclusion, orthopedic surgery patients demonstrated insufficient knowledge but generally positive attitudes and proactive practices regarding VTE prevention and treatment, highlighting the key role of attitude in shaping preventive behaviors. These findings underscore the importance of patient-centered care, in which VTE education and counseling are tailored to patients’ information needs and barriers to adherence. Targeted educational interventions should be integrated into orthopedic care pathways, alongside routine KAP assessment and VTE-focused preoperative counseling, to help bridge the knowledge gap and support evidence-based practice.

## Data Availability

The original contributions presented in the study are included in the article/Supplementary material, further inquiries can be directed to the corresponding author.
