# Supplementary material for: Knowledge, attitude, and practice of orthopedic surgery patients regarding the prevention and treatment of venous thromboembolism
Source: Front Public Health. 2026 Feb 9;14:1676207. doi: 10.3389/fpubh.2026.1676207 (PMC12926403; doi:10.3389/fpubh.2026.1676207)
Supplement: Supplementary file 1 [file Table_1.docx]

**Supplement Table 1: Pilot baseline characteristic**

|  | **N (%)** |
| --- | --- |
|  |  |
| **Total** | 26 |
| **Gender** |  |
| Male | 19 (73.08) |
| Female | 7 (26.92) |
| **Age (years old)** | 48.65 ± 16.67 |
| **Residence** |  |
| Rural | 3 (11.54) |
| Urban | 18 (69.23) |
| Suburban | 5 (19.23) |
| **Education** |  |
| Middle school or below | 13 (50) |
| High school/vocational school | 6 (23.08) |
| Associate degree | 1 (3.85) |
| Bachelor’s degree or above | 6 (23.08) |
| **Average monthly income per capita** |  |
| <2,000 | 2 (7.69) |
| 2,000-5,000 | 14 (53.85) |
| >5,000-10,000 | 10 (38.46) |
| **Type of medical insurance** |  |
| Only social medical insurance | 23 (88.46) |
| Both social and commercial medical insurance | 1 (3.85) |
| No insurance | 2 (7.69) |
| **BMI** |  |
| <18.5 | 3 (11.54) |
| 18.5-23.9 | 12 (46.15) |
| ≥24.0 | 11 (42.31) |
| **Underlying disease** |  |
| Diabetes | 2 (7.69) |
| Hypertension | 6 (23.08) |
| Myocardial infarction | 1 (3.85) |
| None | 17 (65.38) |
| **Part with orthopedic surgery** |  |
| Knee joint | 4 (15.38) |
| Ankle joint | 8 (30.77) |
| Hip joint | 3 (11.54) |
| Shoulder | 3 (11.54) |
| Wrist | 3 (11.54) |
| Spine | 4 (15.38) |
| Elbow joint | 1 (3.85) |
| **Type of orthopedic surgery** |  |
| Arthroscopic surgery | 3 (11.54) |
| Joint replacement | 1 (3.85) |
| Fracture fixation | 12 (46.15) |
| Others | 10 (38.46) |
| **Smoking habits** |  |
| Yes | 14 (53.85) |
| No | 12 (46.15) |
| **Drinking habits** |  |
| Yes | 9 (34.62) |
| No | 17 (65.38) |
| **History of venous thromboembolism** |  |
| Yes | 1 (3.85) |
| No | 25 (96.15) |
| **Medication prevent thrombosis** |  |
| Yes | 11 (42.31) |
| No | 15 (57.69) |
| **Family history of thrombosis** |  |
| No | 26 (100) |
| **Knowledge about venous thromboembolism** |  |
| From a doctor’s explanation | 9 (34.62) |
| Internet, public accounts, short videos | 4 (15.38) |
| No knowledge | 13 (50) |

**Supplementary Table 2. Confirmatory Factor Analysis (CFA) Fit Indices for the Questionnaire**

| Indicator | Reference | Actual |
| --- | --- | --- |
| CMIN/DF | 1–3: Excellent; 3–5: Good | 1.659 |
| RMSEA | <0.08: Good | 0.035 |
| IFI | >0.80: Good | 0.968 |
| TLI | >0.80: Good | 0.965 |
| CFI | >0.80: Good | 0.968 |

**Supplement** **Table 3: Bonferroni-Adjusted Multiple Comparisons for Subgroup Differences in KAP Scores**

|  | | Knowledge | | Attitude | | Practice | |
| --- | --- | --- | --- | --- | --- | --- | --- |
|  |  | P | Bonferroni P | P | Bonferroni P | P | Bonferroni P |
| **Residence** | Rural VS Urban |  |  | <0.001 | <0.001 |  |  |
|  | Rural VS Suburban |  |  | 0.022 | 0.067 |  |  |
|  | Urban VS Suburban |  |  | 0.400 | 0.999 |  |  |
| **Education** | Middle school or below VS High school/vocational school |  |  | 0.080 | 0.478 |  |  |
|  | Middle school or below VS Associate degree |  |  | 0.019 | 0.114 |  |  |
|  | Middle school or below VS Bachelor’s degree or above |  |  | <0.001 | <0.001 |  |  |
|  | High school/vocational school VS Associate degree |  |  | 0.312 | 0.999 |  |  |
|  | High school/vocational school VS Bachelor’s degree or above |  |  | <0.001 | 0.999 |  |  |
|  | Associate degree VS Bachelor’s degree or above |  |  | <0.001 | 0.999 |  |  |
| **Average monthly income per capita** | <2,000 VS 2,000-5,000 |  |  | 0.276 | 0.829 |  |  |
|  | <2,000 VS >5,000-10,000 |  |  | <0.001 | <0.001 |  |  |
|  | 2,000-5,000 VS >5,000-10,000 |  |  | <0.001 | <0.001 |  |  |
| **BMI** | <18.5 VS 18.5-23.9 |  |  |  |  | 0.020 | 0.059 |
|  | <18.5 VS ≥24.0 |  |  |  |  | 0.004 | 0.011 |
|  | 18.5-23.9 VS ≥24.0 |  |  |  |  | 0.250 | 0.750 |
| **Underlying disease** | Diabetes VS Hypertension |  |  | 0.870 | 0.999 |  |  |
|  | Diabetes VS Varicose veins |  |  | 0.606 | 0.999 |  |  |
|  | Diabetes VS Overweight/obesity |  |  | 0.606 | 0.999 |  |  |
|  | Diabetes VS Myocardial infarction |  |  | 0.522 | 0.999 |  |  |
|  | Diabetes VS Malignant tumors |  |  | 0.452 | 0.999 |  |  |
|  | Diabetes VS Rheumatic and autoimmune diseases |  |  | 0.174 | 0.999 |  |  |
|  | Diabetes VS None |  |  | <0.001 | <0.001 |  |  |
|  | Hypertension VS Varicose veins |  |  | 0.776 | 0.999 |  |  |
|  | Hypertension VS Overweight/obesity |  |  | 0.765 | 0.999 |  |  |
|  | Hypertension VS Myocardial infarction |  |  | 0.664 | 0.999 |  |  |
|  | Hypertension VS Malignant tumors |  |  | 0.615 | 0.999 |  |  |
|  | Hypertension VS Rheumatic and autoimmune diseases |  |  | 0.316 | 0.999 |  |  |
|  | Hypertension VS None |  |  | <0.001 | 0.001 |  |  |
|  | Varicose veins VS Overweight/obesity |  |  | 0.975 | 0.999 |  |  |
|  | Varicose veins VS Myocardial infarction |  |  | 0.835 | 0.999 |  |  |
|  | Varicose veins VS Malignant tumors |  |  | 0.791 | 0.999 |  |  |
|  | Varicose veins VS Rheumatic and autoimmune diseases |  |  | 0.400 | 0.999 |  |  |
|  | Varicose veins VS None |  |  | <0.001 | <0.001 |  |  |
|  | Overweight/obesity VS Myocardial infarction |  |  | 0.864 | 0.999 |  |  |
|  | Overweight/obesity VS Malignant tumors |  |  | 0.827 | 0.999 |  |  |
|  | Overweight/obesity VS Rheumatic and autoimmune diseases |  |  | 0.453 | 0.999 |  |  |
|  | Overweight/obesity VS None |  |  | <0.001 | <0.001 |  |  |
|  | Myocardial infarction VS Malignant tumors |  |  | 0.981 | 0.999 |  |  |
|  | Myocardial infarction VS Rheumatic and autoimmune diseases |  |  | 0.631 | 0.999 |  |  |
|  | Myocardial infarction VS None |  |  | <0.001 | 0.008 |  |  |
|  | Malignant tumors VS Rheumatic and autoimmune diseases |  |  | 0.601 | 0.999 |  |  |
|  | Malignant tumors VS None |  |  | <0.001 | 0.001 |  |  |
|  | Rheumatic and autoimmune diseases VS None |  |  | <0.001 | 0.001 |  |  |
| **Knowledge about venous thromboembolism** | From a doctor’s explanation VS lectures, online or offline courses | 0.107 | 0.999 |  |  |  |  |
|  | From a doctor’s explanation VS Internet, public accounts, short videos | 0.359 | 0.999 |  |  |  |  |
|  | From a doctor’s explanation VS Books, literature | 0.553 | 0.999 |  |  |  |  |
|  | From a doctor’s explanation VS No knowledge | <0.001 | <0.001 |  |  |  |  |
|  | lectures, online or offline courses VS Internet, public accounts, short videos | 0.486 | 0.999 |  |  |  |  |
|  | lectures, online or offline courses VS Books, literature | 0.070 | 0.698 |  |  |  |  |
|  | lectures, online or offline courses VS No knowledge | <0.001 | <0.001 |  |  |  |  |
|  | Internet, public accounts, short videos VS Books, literature | 0.202 | 0.999 |  |  |  |  |
|  | Internet, public accounts, short videos VS No knowledge | <0.001 | <0.001 |  |  |  |  |
|  | Books, literature VS No knowledge | <0.001 | <0.001 |  |  |  |  |

**Supplementary table 4. SEM fit indices**

| **Indicators** | **Reference** | **Actual** |
| --- | --- | --- |
| CMIN/DF | 1-3: Excellent, 3-5: Good | 1.659 |
| RMSEA | <0.08: Good | 0.035 |
| IFI | >0.8: Good | 0.968 |
| TLI | >0.8: Good | 0.965 |
| CFI | >0.8: Good | 0.968 |
