# Supplementary material for: Knowledge, attitude, and practice of orthopedic surgery patients regarding the prevention and treatment of venous thromboembolism
Source: Front Public Health. 2026 Feb 9;14:1676207. doi: 10.3389/fpubh.2026.1676207 (PMC12926403; doi:10.3389/fpubh.2026.1676207)
Supplement: Supplementary file 2 [file Table_2.docx]

| **Part 1 Basic Information** |
| --- |

| **Gender** |
| --- |
| Male |
| Female |
| **Age (years old)** |
| **Residence** |
| Rural |
| Urban |
| Suburban |
| **Education** |
| Middle school or below |
| High school/vocational school |
| Associate degree |
| Bachelor’s degree or above |
| **Average monthly income per capita** |
| <2,000 |
| 2,000-5,000 |
| >5,000-10,000 |
| **Type of medical insurance** |
| Only social medical insurance |
| Only commercial medical insurance |
| Both social and commercial medical insurance |
| No insurance |
| **BMI** |
| <18.5 |
| 18.5-23.9 |
| ≥24.0 |
| **Underlying disease** |
| Diabetes |
| Hypertension |
| Varicose veins |
| Overweight/obesity |
| Myocardial infarction |
| Malignant tumors |
| Rheumatic and autoimmune diseases |
| None |
| **Part with orthopedic surgery** |
| Knee joint |
| Ankle joint |
| Hip joint |
| Shoulder |
| Wrist |
| Spine |
| Elbow joint |
| **Type of orthopedic surgery** |
| Arthroscopic surgery |
| Joint replacement |
| Fracture fixation |
| Others |
| **Smoking habits** |
| Yes |
| No |
| **Drinking habits** |
| Yes |
| No |
| **History of venous thromboembolism** |
| Yes |
| No |
| **Medication prevent thrombosis** |
| Yes |
| No |
| **Family history of thrombosis** |
| Yes |
| No |
| **Knowledge about venous thromboembolism** |
| From a doctor’s explanation |
| lectures, online or offline courses |
| Internet, public accounts, short videos |
| Books, literature |
| No knowledge |

| **Part 2** **Knowledge of Venous Thromboembolism** | | | |  |
| --- | --- | --- | --- | --- |
| **1.** **Venous thromboembolism refers to the abnormal clotting of blood in veins, leading to complete or partial blockage of the blood vessel.** | a. True | b. False | c. Not sure | |
| **2.** **Patients undergoing major orthopedic surgeries (e.g., total hip or total knee replacement) are at increased risk of venous thrombosis due to factors like vascular injury and reduced activity.** | a. True | b. False | c. Not sure | |
| **3.** **Deep vein thrombosis and pulmonary embolism are two clinical manifestations of venous thromboembolism occurring in different locations and at different stages.** | a. True | b. False | c. Not sure | |
| **4.** **Pulmonary embolism is a major cause of death during the perioperative period in orthopedic surgery, and 90% of emboli originate from deep vein thrombosis.** | a. True | b. False | c. Not sure | |
| **5.** **Among patients with proximal deep vein thrombosis, 50% typically present with symptomatic or asymptomatic pulmonary embolism.** | a. True | b. False | c. Not sure | |
| **6.** **The most common site for deep vein thrombosis is the upper limb.** | a. True | b. False | c. Not sure | |
| **7.** **Major trauma and orthopedic surgery are risk factors for venous thromboembolism.** | a. True | b. False | c. Not sure | |
| **8.** **If pulmonary embolism is untreated or treatment is delayed, the mortality rate can be as high as 85%, but with active and proper treatment, the mortality rate can drop to 10%.** | a. True | b. False | c. Not sure | |
| **9.** **Basic preventive measures and physical preventive measures alone are sufficient to prevent venous thromboembolism after orthopedic surgery.** | a. True | b. False | c. Not sure | |
| **10.** **For patients with traumatic orthopedic injuries, the risk period for venous thrombosis starts immediately after injury. Thrombosis tendencies may appear within 24 hours of injury, and the high-risk period for thrombosis extends to 35 days post-surgery.** | a. True | b. False | c. Not sure | |
| **11.** **During the acute phase of lower limb deep vein thrombosis, the affected limb should be elevated to relieve swelling, and hot compresses and massage should be applied.** | a. True | b. False | c. Not sure | |
| **12.** **Anticoagulant medications (e.g., heparin) administered before and after major surgeries such as joint replacement can prevent blood clots and reduce the occurrence of pulmonary embolism.** | a. True | b. False | c. Not sure | |

| **Part 3 Attitudes Toward Venous Thromboembolism** | | | | | |
| --- | --- | --- | --- | --- | --- |
| **1.** **You are willing to participate in seminars related to venous thromboembolism. (P)** | a. strongly agree | b. agree | c. neutral | d. disagree | e. strongly disagree |
| **2.** **You are able to consistently follow your doctor’s instructions for taking medication. (P)** | a. strongly agree | b. agree | c. neutral | d. disagree | e. strongly disagree |
| **3.** **If you experience swelling or pain in your limbs after surgery, you will proactively inform healthcare staff. (P)** | a. strongly agree | b. agree | c. neutral | d. disagree | e. strongly disagree |
| **4.** **You believe that untreated venous thrombosis may lead to complications. (P)** | a. strongly agree | b. agree | c. neutral | d. disagree | e. strongly disagree |
| **5.** **You believe that a dislodged thrombus from venous thrombosis will not endanger the patient’s life. (N)** | a. strongly agree | b. agree | c. neutral | d. disagree | e. strongly disagree |
| **6.** **You find it acceptable to use doctor-recommended medications for thrombosis prevention. (P)** | a. strongly agree | b. agree | c. neutral | d. disagree | e. strongly disagree |
| **7.** **You are willing to perform simple functional training using medical equipment. (P)** | a. strongly agree | b. agree | c. neutral | d. disagree | e. strongly disagree |
| **8.** **You are willing to improve your lifestyle to prevent venous thrombosis, such as quitting smoking and drinking, adopting a healthy diet, and controlling blood sugar and lipids. (P)** | a. strongly agree | b. agree | c. neutral | d. disagree | e. strongly disagree |

| **Part 4 Practices for Preventing Venous Thromboembolism** | | | | | |
| --- | --- | --- | --- | --- | --- |
| **Have you engaged in the following behaviors after orthopedic surgery to prevent venous thrombosis?** |  |  |  |  |  |
| **1.** **Elevating the affected limb post-surgery** | a. always | b. often | c. sometimes | d. rarely | e. never |
| **2.** **Performing appropriate functional exercises** | a. always | b. often | c. sometimes | d. rarely | e. never |
| **3.** **Quitting smoking** | a. always | b. often | c. sometimes | d. rarely | e. never |
| **4.** **Quitting alcohol consumption** | a. always | b. often | c. sometimes | d. rarely | e. never |
| **5.** **Maintaining a light diet and controlling blood sugar and lipids** | a. always | b. often | c. sometimes | d. rarely | e. never |
| **6.** **Observing the effects of medication on yourself** | a. always | b. often | c. sometimes | d. rarely | e. never |
