# Supplementary figures and images for: Knowledge, attitude, and practice of orthopedic surgery patients regarding the prevention and treatment of venous thromboembolism
Source: Front Public Health. 2026 Feb 9;14:1676207. doi: 10.3389/fpubh.2026.1676207 (PMC12926403; doi:10.3389/fpubh.2026.1676207)

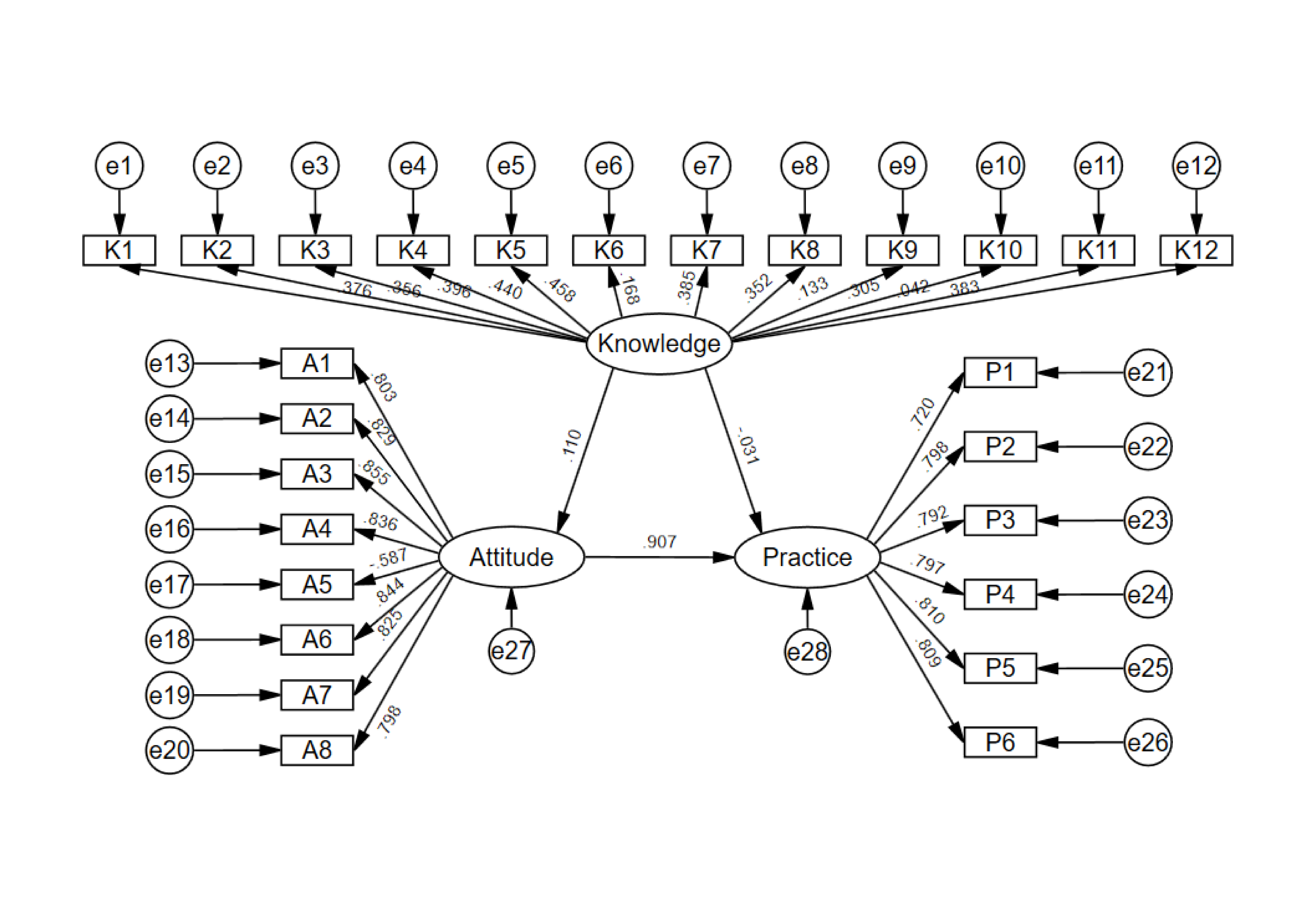

Supplement: Supplementary file 3 [file Image_1.tif]
